# Towards a gendered diabetes care approach: a secondary analysis of conventional and complementary medicine diabetes health service use of older women with diabetes and a mental health co-morbidity

**DOI:** 10.1186/s12906-026-05413-3

**Published:** 2026-07-28

**Authors:** Tracey Oorschot, Jon Adams, David Sibbritt

**Affiliations:** School of Public Health, University of Technology Sydney, PO Box 123 Broadway, Sydney, NSW 2007 Australia

**Keywords:** Women’s health, Diabetes, Mental health, Health service use, Public health

## Abstract

**Background:**

Women with diabetes have different self-management and self-care characteristics and risk profiles to men. A difference of note is that women with diabetes are at greater risk of anxiety or depression. However, limited research has explored if the presence of a mental health co-morbidity is an influencing factor upon health service use pertinent to diabetes self-care.

**Materials and methods:**

A secondary analysis of a chronic disease self-care sub-study stemming from the 45 and Up Study was conducted. Cross-sectional analyses were completed to examine the conventional and complementary medicine health service use patterns of women with diabetes and anxiety or depression, or anxiety and depression (*n* = 451, *n* = 439, *n* = 439, respectively).

**Results:**

Of the women who completed the diabetes specific survey and mental health questions, 155 (35.3%) were classified as clinically depressed, 94 (20.8%) reported anxiety and 58 (13.2%) were classified as having both depression and anxiety. A regression analysis revealed women with co-morbid depression were the most likely to consult with their general practitioner 7 or more times annually (OR: 3.07,95% CI 1.35–6.89), those with co-morbid anxiety most likely to engage in yoga or meditation practice (OR: 4.37, 95% CI 2.05–9.32), and those with co-morbid depression *and* anxiety most likely to consult with a counsellor or psychologist (OR: 7.41, 95% CI 3.22–17.07).

**Conclusion:**

Our findings reveal the need for a diverse range of health care professionals, both within conventional and complementary medicine, to engender coordinated and targeted diabetes care with a view to supporting the specific needs of women with diabetes.

## Background

Diabetes mellitus is one of many known age-associated chronic conditions [1]. Globally, most diagnoses of diabetes occur within the 45 to 64 age group, with prevalence steadily increasing from around 11% for 45–49 years to around 20% for over 65 year olds [2]. Consequently, preventive action has increased in prominence and has adopted a two-fold approach – targeted health promotion to younger cohorts to prevent diabetes onset and for older cohorts already living with diabetes, increased attention on supporting diabetes self-management. Supporting diabetes self-management has become a critical diabetes care strategy to prevent diabetes progression and onset of co-morbidities that contribute to morbidity and mortality [3]. Diabetes self-management typically refers to an individual’s regular diabetes care routines including blood glucose monitoring, diet, exercise and use of diabetes therapies, and so on [4]. However, another key component of diabetes self-management pertains to supporting and encouraging self-care which includes help-seeking and health service use that promotes diabetes care [3, 4]. Diabetes self-care strategies are known to be diverse, with previous research revealing gender differences.

Gender differences have been identified in diabetes self-care attitudes, beliefs, behaviours, motivations and concerns [7, 8]. This includes differentiating concerns about the impacts connected with caring for family, such as women being more concerned about *caring* for family, while men report being more concerned about *providing* for family [7, 8]. Women were also more likely to report diabetes as a negative life impact than men [7]. Other areas where gender differences have been reported relate to: the capacity for self-care (e.g. women being more likely to have disrupted capacity due to caring responsibilities, while men report being impacted more by work responsibilities); help-seeking behaviours (e.g. women being more likely to seek professional health care or social support than men); and health service use (e.g. women being more likely to access diabetes health services and to consult with a higher number of concurrent health care professionals (HCPs) than men) [8].These findings reveal the need for multi-disciplinary diabetes care teams to respond to divergent care needs at the individual level [5, 6].

Lastly, diabetes complications and co-morbidities were also found to have gender differences. For example, women have been found to have a greater risk of mortality from either cardiovascular disease or stroke than men, and are more likely to report anxiety or depression than men [7, 9, 10], with mental health conditions in turn resulting in gendered mortality risks [11]. Within Australia, current diabetes clinical guidelines do refer to gendered physiological risk profiles but fail to mention psychological risks. Whilst a need to review mental health care as part of diabetes care is broadly mentioned, the Australian National Diabetes Strategy 2021–2030, also makes no reference to gender differences in diabetes care, again aside from obvious references to gestational diabetes, and pre-conception/post-partum issues [12]. Furthermore, past research focused upon those living with diabetes has shown women are more likely to use complementary medicine (CM) (including product and practice use and consultation with CM practitioners) compared to men, with this use typically concurrent to conventional medicine [13, 14]. This use is known to have clinical governance concerns [15, 16] and is also not mentioned in current clinical guidelines or health policies. This is despite the existence of safe and patient-centred health care practice resources developed by the Government funded National Health and Medical Research Council to support clinicians to have conversations with patients about complementary medicine use [17]. This reveals a need for gendered diabetes research to address current clinical guideline and policy gaps, particularly in relation to diabetes-related mental health care.

The need for a gendered approach to diabetes care and in particular how to incorporate and respond to mental health care needs is evident. Whilst the higher prevalence of anxiety or depression for women generally is gaining increasing research attention in relation to diabetes care, gender differences remain under-researched [7, 8, 15]. Additionally, although it is broadly known that the presence of any mental health condition in the context of diabetes can lead to avoidance, withdrawal or delayed engagement with health services, and poorer health outcomes [18], we know relatively little about the role of a common co-morbidity such as anxiety or depression as an influencing factor upon health care service use choices. Unfortunately, no study to date has examined the influence of mental health status upon conventional and CM health service use amongst women with diabetes. The research reported here focuses on the health service use patterns of women to begin addressing gendered diabetes care knowledge gaps by providing empirical insights to help ensure better coordinated diabetes care for women. Understanding how the presence of anxiety or depression may lead to engagement or disengagement with specific HCPs, products or practices can assist health care professionals in meeting the specific needs of women with diabetes, alongside supporting clinical practice and health care policy development to support gendered diabetes cares overall.

## Materials and methods

This paper discusses the results from a secondary analysis of a diabetes self-care sub-study stemming from The Sax Institute’s 45 and Up Study. The 45 and Up Study was established in 2006, with 267,153 participants surveyed between 2006 and 2009. Participants were randomly sampled from the Services Australia (formerly the Australian Government Department of Human Services) Medicare enrolment database consisting of males and females aged from 45 years who were residents of New South Wales, equating to a response rate of around 18% [19]. The sub-study was conducted between September and December 2016. Of the 4,500 women who indicated a diabetes diagnosis in the 45 and Up Study, 800 women were randomly invited to participate in the diabetes specific questionnaire sub-study utilising simple random sampling. The diabetes specific sub-study was completed by 451 respondents reporting a diagnosis of either Type 1 or 2 diabetes, with an age range from 53 to 95 (M = 69.49, SD = 8.51). Tables 1, 2 and 3 report the final sample sizes of the women who completed both the diabetes specific survey and the questions regarding mental health. This equated to a final sample size of *n* = 439 for the depression group, *n* = 451 for the anxiety group, and *n* = 439 for the depression and anxiety group. Ethics approval for the 45 and Up Study was obtained by the University of New South Wales Human Research Ethics Committee. Ethics approval for the sub-study was obtained by the Human Research Ethics Committee at the University of Technology Sydney.

**Table 1:** Socio-demographic characteristics by mental health status

Characteristic	MH Status								
	Depression (*n* = 439)			Anxiety (*n* = 451)			Depression & Anxiety (*n* = 439)		
	No (*n* = 284)	Yes (*n* = 155)	*p*-value	No (*n* = 357)	Yes (*n* = 94)	*p*-value	No (*n* = 381)	Yes (*n* = 58)	*p*-value
	n (%)	n (%)		n (%)	n (%)		n (%)	n (%)	
Socio-demographic									
*Area of residence*			*0.581*			*0.498*			*0.572*
Major cities	117 (42.5)	70 (46.0)		148 (42.8)	46 (49.5)		159 (43.0)	28 (49.1)	
Inner regional	114 (41.4)	63 (41.4)		147 (42.5)	34 (36.6)		157 (42.4)	20 (35.1)	
Outer reg/remote	44 (16.0)	19 (12.5)		51 (14.7)	13 (13.9)		54 (14.6)	9 (15.8)	
*Marital Status*			*0.05*			*0.272*			*0.596*
Single	16 (5.7)	9 (5.9)		21 (5.9)	5 (5.3)		23 (6.1)	2 (3.4)	
Married/de facto	187 (66.3)	84 (54.9)		224 (63.5)	52 (55.3)		236 (62.6)	35 (60.3)	
Separated/divorced/widowed	79 (28.0)	60 (39.2)		108 (30.6)	37 (39.4)		118 (31.3)	21 (36.2)	
*Highest Qualification*			*0.324*			*0.916*			*0.582*
No school/school/high school cert	132 (46.9)	82 (53.2)		175 (49.4)	45 (48.4)		183 (48.4)	31 (54.4)	
Trade/Tertiary cert/diploma	80 (28.5)	43 (27.9)		99 (27.9)	28 (30.1)		107 (28.3)	16 (28.1)	
University degree	69 (24.6)	29 (18.8)		80 (22.6)	20 (21.5)		88 (23.3)	10 (17.5)	
*Financial situation*			*< 0.001*			*0.011*			*< 0.001*
No or little difficulties	192 (68.1)	66 (42.6)		223 (62.8)	43 (45.7)		241 (63.6)	17 (29.3)	
Some difficulties	69 (24.5)	42 (27.1)		83 (23.4)	31 (33.0)		89 (23.5)	22 (37.9)	
Struggles with income	21 (7.4)	47 (30.3)		49 (13.8)	20 (21.3)		49 (12.9)	19 (32.8)	
*PHI status* ^*a*^			*< 0.001*			*0.382*			*0.088*
No	92 (32.9)	188 (67.1)		137 (38.9)	40 (44.0)		146 (38.8)	28 (50.9)	
Yes	82 (54.3)	69 (45.7)		215 (61.1)	51 (56.0)		230 (61.2)	27 (49.1)	

a: PHI hospital = private health insurance coverage for treatment services in Australian hospitals by a private health care provider

### Mental health status

Mental health status was grouped by depression, anxiety or depression and anxiety. The results from the Center for Epidemiologic Depression Scale (CESD-10: [20]) contained within the sub-study were used to determine depression status. Women with a score of 10 or more were deemed as depressed (yes), with those with a score under 10 deemed not depressed (no), in line with the convention of applying a threshold of 10 to reflect clinical depression. Respondents were asked to indicate diagnosis within the last 12 months of a variety of health conditions, such as mental health status. Women who self-reported an anxiety disorder were deemed as having anxiety (yes), with those both self-reporting anxiety and meeting the criteria for clinical depression classified as having both depression and anxiety (yes).

### Health service use

Participants were asked if they consulted with conventional or CM practitioners, as well as practices or products directly in relation to their diabetes care within the last 12 months. Questions included frequency of visit to conventional practitioners such as a general practitioner (GP) (primary care), medical specialist e.g. endocrinologist (secondary care), hospital doctor (tertiary care) or allied health (e.g. nurse, pharmacist, psychologist). Examples of CM practitioners included naturopath, massage therapist, chiropractor and osteopath. Consultation with either a pharmacist or chemist were combined into the one practitioner group, as were consultation with either a counsellor or psychologist, or naturopath or herbalist. GP consultation frequency were arranged into three sub-groups – 1–2 (low), 3–6 (med) or 7 or more (high) consultations annually. Conventional practices or products included prescription medicine use (both diabetes and non-diabetes pharmaceuticals), whilst CM practices or products included herbal medicines, meditation or yoga, multivitamins or aromatherapy. Similarly, use of yoga or meditation as a self-care practice were combined to create one practice group – yoga/meditation.

### Statistical analyses and covariates

Stata, version 16.1 [21] was utilised to perform all statistical analyses. Explanatory variables corresponded with mental health status, whilst conventional or CM health service use were adopted as outcome variables. Prevalence of health service use was calculated by use of two-way tables, with chi-squared tests (and Fisher’s exact test where required) undertaken to ascertain significant associations between mental health status and health service use. Logistic regression modelling was undertaken to determine likelihood of health service use according to mental health status, with likelihood ratio testing used to establish statistical significance of the overall model. The models were adjusted for several socio-demographic variables including area of residence, marital status, highest qualification and financial situation. An alpha level of 0.05 was set to indicate statistical significance.

## Results

### Socio-demographic characteristics by mental health status

Most women met the criteria for clinical depression (35%, *n* = 155), whilst 20.8% (*n* = 94) reported anxiety and 13.2% (*n* = 58) were established to have both depression & anxiety. Socio-demographic characteristics are shown in Table 1. Reporting the highest level of financial difficulty (struggles with income) was significantly (*p* < 0.05) associated with any mental health status, with those with depression and anxiety reporting the greatest proportion. By contrast women without a mental health co-morbidity predominantly reported no or little financial difficulty. Possessing private health insurance was significantly (*p* < 0.05) associated with depression only, with those with depression less likely to hold private insurance compared to those without depression.

### Prevalence of health service use of women with diabetes by mental health status

As shown in Table 2, consulting with either a counsellor or psychologist was significantly (*p* < 0.05) associated across all mental statuses, with those with depression & anxiety having the highest consultation rate. For women with depression, significant (*p* < 0.05) associations were found in relation to GP, pharmacy/chemist, and occupational therapy consultations.

**Table 2:** Prevalence of Health Service Use of women with diabetes mellitus by mental health status

Health Service Use (yes/no)		MH Status								
		Depression (*n* = 439)			Anxiety (*n* = 451)			Depression & Anxiety (*n* = 439)		
		No (*n* = 284)	Yes (*n* = 155)	*p*-value	No (*n* = 357)	Yes (*n* = 94)	*p*-value	No (*n* = 381)	Yes (*n* = 58)	*p*-value
		n (%)	n (%)		n (%)	n (%)		n (%)	n (%)	
Practitioners										
*Conventional*										
*GP*	Low	115 (49.8)	46 (34.6)	*< 0.001*	135 (46.1)	32 (39.5)	*0.538*	145 (46.1)	16 (32.0)	*0.092*
	Med	98 (42.4)	59 (44.4)		122 (41.7)	39 (48.1)		133 (42.4)	24 (48.0)	
	High	18 (7.8)	28 (21.0)		36 (12.3)	10 (12.3)		36 (11.5)	10 (20.0)	
*Medical Specialist*	yes	54 (19.0)	48 (31.0)	*0.005*	84 (23.5)	23 (24.5)	*0.849*	83 (21.8)	19 (32.8)	*0.065*
	no	230 (51.0)	107 (69.0)		273 (76.5)	71 (75.5)		298 (78.2)	39 (67.2)	
*Hospital doctor*	yes	16 (5.6)	16 (10.3)	*0.071*	25 (7.0)	10 (10.6)	*0.241*	27 (7.1)	5 (8.6)	*0.675*
	no	268 (94.4)	139 (89.7)		332 (93.0)	84 (89.4)		354 (92.9)	53 (91.4)	
*Nurse*	yes	55 (19.4)	34 (21.9)	*0.522*	75 (21.0)	16 (17.0)	*0.391*	79 (20.7)	10 (17.2)	*0.538*
	no	229 (80.6)	121 (78.1)		282 (79.0)	78 (83.0)		302 (79.3)	48 (82.8)	
*Pharmacist/chemist*	yes	60 (21.1)	47 (30.3)	*0.032*	91 (25.5)	21 (22.3)	*0.529*	93 (24.4)	14 (24.1)	*0.964*
	no	224 (78.9)	108 (69.7)		266 (74.5)	73 (77.7)		288 (75.6)	44 (75.9)	
*Counselor/psychologist*	yes	10 (3.5)	26 (16.8)	*< 0.001*	18 (5.0)	19 (20.2)	*< 0.001*	21 (5.5)	15 (25.9)	*< 0.001*
	no	274 (96.5)	129 (83.2)		339 (95.0)	75 (79.8)		36 (94.5)	43 (74.1)	
*Dietitian*	yes	41 (14.4)	32 (20.7)	*0.095*	61 (17.1)	15 (16.0)	*0.795*	61 (16.0)	12 (20.7)	*0.373*
	no	243 (85.5)	123 (79.3)		296 (82.9)	79 (84.0)		320 (84.0)	46 (79.3)	
*Physiotherapist*	yes	18 (6.3)	16 (10.3)	*0.136*	26 (7.3)	10 (10.7)	*0.286*	26 (6.8)	8 (13.8)	*0.064*
	no	266 (93.7)	139 (89.7)		331 (92.7)	84 (89.3)		355 (93.2)	50 (86.2)	
*Occupational therapist*	yes	* (1.1)	12 (7.7)	*< 0.001*	11 (3.1)	5 (5.3)	*0.297*	10 (2.6)	5 (8.6)	*0.019*
	no	281 (98.9)	143 (92.3)		346 (96.9)	89 (94.7)		371 (97.4)	53 (91.4)	
*Complementary Medicine*										
*Acupuncturist*	yes	* (1.4)	* (1.3)	*1.000*	* (1.1)	* (2.1)	*0.609*	5 (1.3)	* (1.7)	*0.801*
	no	280 (98.6)	153 (98.7)		353 (98.9)	92 (97.8)		376 (98.7)	57 (98.3)	
*Chiropractor*	yes	12 (4.2)	5 (3.2)	*0.604*	16 (4.5)	* (2.1)	*0.300*	16 (4.2)	* (1.7)	*0.363*
	no	272 (95.7)	150 (96.8)		341 (95.5)	92 (97.9)		365 (95.8)	57 (98.3)	
*Naturopath/herbalist*	yes	* (1.1)	* (1.9)	*0.431*	5 (1.4)	* (1.1)	*0.800*	6 (1.6)	* (0)	*0.336*
	no	281 (98.9)	152 (98.1)		352 (98.6)	93 (98.9)		375 (98.4)	58 (100)	
*Massage therapist*	yes	17 (6.0)	8 (5.2)	*0.722*	19 (5.3)	6 (6.4)	*0.689*	20 (5.3)	5 (8.6)	*0.302*
	no	267 (94.0)	147 (94.8)		338 (94.7)	88 (93.6)		361 (94.7)	53 (91.4)	
*Nutritionist*	yes	5 (1.8)	5 (3.2)	*0.325*	7 (2.0)	* (3.2)	*0.471*	7 (1.8)	* (5.2)	*0.113*
	no	279 (98.2)	150 (96.8)		350 (98.0)	91 (96.8)		374 (98.2)	55 (94.8)	
*Osteopath*	yes	* (1.4)	* (1.9)	*0.702*	* (1.1)	* (3.2)	*0.161*	* (1.0)	* (5.2)	*0.020*
	no	280 (98.6)	152 (98.1)		353 (98.9)	91 (96.8)		377 (99.0)	55 (94.8)	
*Traditional* *Chinese medicine*	yes	* (0)	* (0.7)	*0.353*	* (0)	* (1.1)	*0.208*	* (0)	* (1.7)	*0.010*
	no	284 (100)	154 (99.3)		357 (100)	93 (98.9)		381 (100)	57 (98.3)	
Practice or Products *Conventional*										
*Prescription Medicine* (diabetes)	yes	199 (82.9)	114 (89.8)	*0.078*	253 (83.5)	67 (91.8)	*0.074*	272 (84.2)	41 (93.2)	*0.115*
	no	41 (17.1)	13 (10.2)		50 (16.5)	6 (8.2)		51 (15.8)	* (6.8)	
*Prescription medicine* (Non-diabetes)	yes	217 (83.8)	113 (83.7)	*0.984*	263 (82.2)	74 (87.1)	*0.286*	285 (83.3)	45 (86.5)	*0.559*
	no	42(16.2)	22 (16.3)		57 (17.8)	11 (12.9)		57 (16.7)	7 (13.5)	
*Complementary Medicine*										
*Aromatherapy oils*	yes	8 (2.8)	* (2.6)	*0.885*	7 (2.0)	5 (5.3)	*0.072*	10 (2.6)	* (3.4)	*0.720*
	no	276 (97.2)	151 (97.4)		350 (98.0)	89 (94.7)		371 (97.4)	56 (96.6)	
*Herbal medicines*	yes	11 (3.9)	5 (3.2)	*0.729*	13 (3.7)	* (3.2)	*0.834*	15 (3.9)	* (1.7)	*0.402*
	no	273 (96.1)	150 (96.8)		344 (96.4)	91 (96.8)		366 (96.1)	57 (98.3)	
*Yoga/meditation * *by self*	yes	21 (7.4)	14 (9.0)	*0.545*	18 (5.0)	17 (18.1)	*< 0.001*	26 (6.8)	9 (15.5)	*0.023*
	no	263 (92.6)	141 (90.9)		339 (95.0)	77 (81.9)		364 (93.2)	49 (84.5)	
*Multivitamins*	yes	41 (14.4)	27 (17.4)	*0.409*	53 (14.8)	16 (17.0)	*0.602*	56 (14.7)	12 (20.7)	*0.240*
	no	243 (85.6)	128 (82.6)		304 (85.1)	78 (83.0)		325 (85.3)	46 (79.3)	
*Chromium*	yes	19 (6.7)	11 (7.1)	*0.872*	22 (6.2)	8 (8.5)	*0.416*	27 (7.1)	* (5.2)	*0.590*
	no	265 (93.3)	144 (92.9)		335 (93.8)	86 (91.5)		354 (92.9)	55 (94.8)	
*Cinnamon*	yes	34 (12.0)	21 (13.5)	*0.633*	49 (13.7)	9 (9.6)	*0.285*	49 (12.9)	6 (10.3)	*0.590*
	no	250 (88.0)	134 (86.5)		308 (86.3)	85 (90.4)		332 (87.1)	52 (89.7)	

*Cells marked with an asterisk have *n* < 5

GP consultations were predominantly between 3 and 6 visits annually for those with depression, whilst those without depression mostly visited their GP once or twice a year. Similarly, those without depression were less likely to visit a pharmacist or occupational therapist than those with depression. Consulting an occupational therapist was also associated with depression & anxiety, with a slightly higher consultation rate than those with depression.

The only significant (*p* < 0.05) CM practitioner group pertained to osteopath consultations in association with depression & anxiety. Those with depression & anxiety had a higher consultation rate than those without. In relation to practice or product use, yoga or meditation practice was significantly (*p* < 0.05) associated with anxiety or depression & anxiety, with women with anxiety having the higher consultation rate.

### Likelihood of health service use by mental health status

Considering likelihood of health service use (see Table 3), women with any mental health co-morbidity were mostly likely to consult with a counsellor or psychologist, with women with depression & anxiety having the highest likelihood of consultation. Women with depression were three times as likely to see their GP 7 or more times annually, whereas women with either anxiety or depression & anxiety were around 3 to 4 times likely to practice yoga or meditation. Conversely, women with anxiety were less likely to consult with a pharmacist.

**Table 3:** Likelihood of health service use by mental health status

Health Service Use (yes)	Mental Health Status								
	Depression ^a^ (*n* = 439)			Anxiety ^a^ (*n* = 451 )			Depression & Anxiety ^a^ (*n* = 439)		
	OR	95% CI	*p*-value	OR	95% CI	*p*-value	OR	95% CI	*p*-value
*Practitioners*									
GP: Med (low) High	1.41	0.805, 2.457	*0.231*		x		x		
	3.07	1.355, 6.891	*0.007*		x			x	
Counselor/ psychologist	4.54	1.366, 6.891	*0.001*	6.93	3.124, 15.380	*< 0.001*	7.24	3.138, 16.689	*< 0.001*
Pharmacist	x			0.47	0.245, 0.918	*0.027*	x		
*Practice or Products*									
Yoga/meditation		x		4.37	2.046, 9.321	*< 0.001*	2.80	1.080, 7.241	*0.034*

a: adjusted for – area of residence, marital status, highest qualification, financial situation, PHI status (private health insurance hospital or ancillary)

## Discussion

Although it is known that a mental health co-morbidity influences diabetes self-management practices, diabetes self-management research predominantly focuses on blood glucose monitoring behaviour, diet, exercise and other lifestyle self-management practices, rather than examination of related diabetes self-care elements such as health service use. Our study is novel in so far that it considers both mental health status and resultant health service use inclusive of *both* conventional medicine and CM. Our results suggest that a mental health co-morbidity is an important characteristic of women’s diabetes self-care behaviours in determining health care choices. Women with anxiety were shown to be more likely to engage in *both* conventional medicine and CM use, whereas women with depression were more likely to utilise conventional health care only. This supports emerging research which shows women with diabetes and a mental health co-morbidity use different health services than those without a mental health co-morbidity [22]. This differentiation of health service use reflects divergent diabetes care needs, with concurrent conventional and CM health care use also carrying implications regarding the need for coordinated clinical diabetes care between conventional medicine and CM practitioners.

Our regression analyses revealed all mental health groups to be significantly associated with consultation with either a counsellor or psychologist, although women with anxiety were more likely than women with depression to engage with these practitioner groups. Furthermore, women with depression were the only group to be significantly associated with consulting with their GP. People with diabetes are known to have high consultation rates with their GP as part of standard diabetes management [23], with a subsequent reasonable expectation to find significant results for GP consultations across all mental health groups not just those with depression. Given this, the implication of our finding is that it suggests women with co-morbid depression have a greater need to consult with a GP when compared to women with anxiety. Such variations in provider use are unsurprising in so far that depression has a different risk profile to anxiety (i.e. suicide) and previous work shows GPs typically treat more patients with depression than allied health practitioners, such as counsellors or psychologists [24–26]. This known differentiation of consultation patterns between GPs and counsellors or psychologists, also likely explains the higher rate of consultation for women with co-morbid anxiety with either a counsellor or psychologist, than those with depression. What is significant is that our findings show that mental health status is likely to be an influencing factor underpinning the health care choices of women with diabetes.

The decreased likelihood of consultation with a pharmacist for women with co-morbid anxiety is somewhat surprising given that pharmacy engagement for any person living with diabetes is common [27, 28]. Additionally, people with any mental health condition also frequently consult with a pharmacist, particularly in relation to seeking advice regarding the use of multiple pharmaceuticals which is a common occurrence for people with multimorbidity [29]. Likewise people with either depression or anxiety can both have reduced help-seeking behaviours [30], which might help explain the reduction in likelihood of consultation with this practitioner group. Further investigation regarding pharmacist consultations for women with diabetes is required to explore if the presence of anxiety (or any mental health issue) is impacting engagement with this practitioner group.

Overall, our lack of significant findings regarding CM use was unexpected, given that women who use CM tend to be older and have chronic conditions [13, 14, 31], which is our core sample for this study. Our analysis did not identify any significant associations for consultation with CM practitioners or use of CM products, which is unusual given women with either diabetes [32–34] or anxiety or depression [35–37] are known to consult with a range of CM practitioners, as well as use a range of CM products. However, we did find a highly significant association between women with co-morbid anxiety and the practice of yoga or meditation which is consistent with previous results indicating that this is a common form of practice undertaken by people with either anxiety [38, 39] or diabetes [40, 41]. People with diabetes report incorporating yoga and/or meditation practice as part of diabetes self-care routines for several reasons. Some of these reasons include seeking stress reduction which in turn promotes glycemic control [42, 43]; yogic practice for either exercise or as a method for improvement in flexibility, strength or posture to aid in capacity for exercise and thereby promoting weight management which is crucial for people with diabetes [40, 41, 44]; improved sleep which is also associated with improved glycemic control [45, 46]; and to improve or maintain general health to reduce risks for common diabetes complications such as cardiovascular disease [40, 47, 48]. This result suggests that women with anxiety might be more likely to include a CM practice as part of their diabetes self-care routines, as opposed to women with depression. The clinical implication of this finding is that women with diabetes and anxiety who are having difficulties with common diabetes self-management practices related to sleep, exercise or blood glucose control, may have improved outcomes by considering the inclusion or either yoga or meditation as part of their diabetes self-care routines. Women with both diabetes and depression may require a different clinical approach.

A strength of this study is that participants were asked directly about what practitioners, practices and products they engaged with as part of their diabetes self-care routines, increasing the validity of our findings in terms of attribution to diabetes self-management behaviours specifically, rather than general self-care behaviours. However, a limitation of the study design is that the questions were not designed specifically to reveal the influence of mental health status upon health care choices. The 45 and Up sample is also not nationally representative, was skewed towards higher income groups, used oversampling of people aged 80 and over and people living in rural and remote areas, and relies upon self-report which can carry risks of recall bias, making it difficult to generalise our findings to the wider diabetes population. It is also important to note that the data was collected in 2016. Due to small sample sizes, attention could not be given to understanding the role of cultural factors or ethnicity; or specific diabetes diagnosis (i.e. Type 1 Diabetes versus Type 2 diabetes), severity, treatment type (e.g. insulin versus non-insulin diabetes therapies) and may also account for some of the non-statistically significant health service use results reported. Nonetheless, gender-based diabetes health services research is limited, and our study based upon analyses drawing from an established database provides valuable insights to the examination and understanding of health care choices of women with diabetes and co-morbid depression or anxiety.

## Conclusions

Despite some of our analyses returning unexpected results, our study augments previous research that indicates that the mental health status of women with diabetes is an influencing factor driving conventional medicine and CM health service use choices. Given the lack of gender-specific diabetes research, further gender-delineated research is indicated to better understand what commonalties and differentiations exist in terms of the conventional and CM health service use choices of men and women with diabetes. This will also provide opportunity to further refine diabetes care and support clinical practice to respond to the diabetes care needs of specific cohorts, such as women with diabetes in light of our overall findings.

Our findings also support the need to consider the role of CM practitioners as it was evident that CM was a choice of diabetes self-care practice for women with anxiety. However, given substantial research indicates that women with any chronic health condition are high users of either CM practitioners or products, and our study did not replicate this, further research examining the CM health service use behaviours of women with diabetes and a mental health co-morbidity is also suggested. Of particular use would be understanding the influence of diabetes as opposed to mental health status as a driver of use or non-use of CM practitioners, products or practices.

Overall, our findings suggest that women’s diabetes care is likely to benefit from a coordinated approach drawing from both conventional medicine and CM HCPs. An integrated and coordinated diabetes care team designed to address the specific needs of women with diabetes and/or a mental health co-morbidity serves multiple purposes. Primarily, it reflects a person-centred rather than provider-centred approach to diabetes care. Person-centred approaches encourages individuals living with diabetes to be actively involved in their own diabetes care, which increases the likelihood of better health and well-being outcomes. From a clinical perspective, it enables inclusion of CM HCPs to support clinical governance of known risks of CM use, as well as providing educational opportunities for conventional medicine HCPs to understand and respond to any benefits or risks from CM use in relation to diabetes and/or mental health care. Finally from a public health perspective, it provides opportunities to collect and analyse valuable health service use data and practice insights to inform clinical practice guidelines and health policy development for gender-specific diabetes care. Best practice integrated care models and how best to include and respond to the mental health care needs of women with diabetes can also be developed.

## Data Availability

The datasets used and/or analysed during the current study are available from the Sax Institute, on reasonable request.
